# Profile and Outcomes of Hospitalized COVID-19 Patients during the Prevalence of the Omicron Variant According to the Brazilian Regions: A Retrospective Cohort Study from 2022

**DOI:** 10.3390/vaccines11101568

**Published:** 2023-10-05

**Authors:** Pedro Dutra Drummond, Daniel Bortot de Salles, Natália Satchiko Hojo de Souza, Daniela Carine Ramires Oliveira, Daniel Ludovico Guidoni, Fernanda Sumika Hojo de Souza

**Affiliations:** 1 Department of Computing, Federal University of Ouro Preto, Morro do Cruzeiro Campus, Ouro Preto 35400-000, MG, Brazil; 2 Laboratory of Immunopathology, Oswaldo Cruz Foundation—Minas, Av. Augusto de Lima 1715, Belo Horizonte 30190-002, MG, Brazil; 3 Department of Mathematics and Statistics, Federal University of São João del-Rei, Praça Frei Orlando 170, São João del Rei 36307-352, MG, Brazil

**Keywords:** COVID-19 vaccines, variants of concern, vaccination, outcomes, Brazil

## Abstract

We investigated the clinical–epidemiological profile and outcomes of COVID-19 patients hospitalized in 2022, during the Omicron variant/subvariant prevalence, in different Brazilian regions to identify the most vulnerable subgroups requiring special attention. Data from COVID-19 patients were extracted from the national Information System for Epidemiological Surveillance of Influenza (SIVEP-Gripe database), and analyses stratified by region and age group were conducted. The constructed dataset encompassed clinical–epidemiological information, intensive care unit admission, invasive and non-invasive ventilation requirements, vaccination status, and evolution (cure or death). It was observed that there were significant differences in the vaccination rates between regions, in the occurrence of unfavorable outcomes, and in the pattern of comorbidities in young patients. The north region had higher rates of unvaccinated patients and a lower percentage of those vaccinated with three doses in all age groups compared to other regions. The northeast region had the highest rates of patients admitted to the ICU for all age groups, while the north and northeast were the most affected by IMV requirements and in-hospital death in all age groups. This study showed that extended vaccination coverage, especially booster doses, can protect different population segments from developing severe disease since lower vaccination coverage was observed in regions with higher fatality rates.

## 1. Introduction

The emergence of new variants of SARS-CoV-2, the etiological agent of COVID-19, has caused new waves of the disease [1]. After the first wave that prevailed throughout 2020, Brazil experienced a second wave due to the emergence of the Gamma/P.1 variant (B.1.1.28.1), which was detected in the Amazon in January 2021, and is more transmissible and lethal than the ancestral coronavirus reported in Wuhan (China). The Gamma/P.1 variant quickly became predominant in Brazil, in the context of a low percentage of vaccinated population [2], whose scheme initially prioritized the vaccination of risk groups, such as the elderly and health workers.

The reduction in the effectiveness of vaccines after 4–6 months [3,4] again increased the risk of fully vaccinated people (two doses/single dose), especially elderly people who were the first to be vaccinated, such that a booster dose was recommended in September 2021 in Brazil [5]. The Omicron variant and subvariants, with numerous mutations, are more transmissible, infective, and capable of evading immunity induced by vaccines or previous infection [6,7], and became dominant in several countries, including Brazil [8]. Omicron caused a third wave, whose peak occurred in March 2022. Then, a new, less intense wave occurred due to the emergence and dominance of the Omicron BA.4/BA.5 subvariants. Therefore, throughout 2022 there was a predominance of the Omicron variant/subvariants in Brazil [9]. Due to the growing number of cases and the absence of specific vaccines for variants, in June 2022, the Ministry of Health recommended a fourth dose (second booster) of vaccine for health workers and individuals over 50 years old. In this context, although mass vaccination has reduced the COVID-19 incidence rate, the drop in immunity, variant vaccine escape, and low vaccination coverage of booster doses made it challenging to control new disease outbreaks throughout 2022.

This highly dynamic temporal process of COVID-19 has suggested a variation in the subgroups most prone to an unfavorable clinical outcome according to the prevalence of the variant, vaccination status, age group, and pre-existing medical conditions (comorbidities) in individuals with COVID-19. During the second wave (Gamma/P.1 variant prevalence), a significant increase in the number of hospitalizations, intensive care unit admissions, and deaths of younger patients (20–59 years) was observed in Brazil as a whole [10]. In that period, a study showed that there was a significant increase in daily cases of younger people (20–59 years old) being hospitalized, admissions to the intensive care unit, invasive mechanical ventilation, and in-hospital deaths, but with a wide variation in the dynamics of occurrences according to the federative unit [11].

Preliminary data have shown that even individuals with a complete vaccination schedule can become infected and develop symptomatic COVID-19 (vaccine breakthrough infection) [12,13,14], since the vaccines currently in use were produced against the ancestral coronavirus discovered in Wuhan (China) and not for variants that are in circulation [1]. The reduction of vaccine-induced immunity over time is another factor that can lead to a breakthrough vaccine infection. A study involving Brazilian patients hospitalized during the prevalence of the P.1 variant showed that elderly people with comorbidities, with a complete vaccination schedule but without a booster dose, are more vulnerable to breakthrough infection (vaccine escape). Admission to the intensive care unit and the requirement of invasive mechanical ventilation (IMV) were factors indicative of increased risk for mortality [15].

Considering that the scenario changed during the pandemic regarding preventive measures, the emergence of new variants of concern, waning immunity, and the coverage and effectiveness of monovalent vaccines in use [16], we proposed to investigate the clinical–epidemiological profile and unfavorable outcomes of COVID-19 patients hospitalized during the prevalence of the Omicron variant/subvariants according to the vaccine status, in addition to analyzing the risk factors for in-hospital death by region, in order to show possible differences among Brazilian regions and identify more vulnerable subgroups that require appropriate care. This exploratory study analyzed records of 115,638 Brazilian COVID-19 patients hospitalized during 2022, stratified by region and age group. The vaccination profile of the patients and the primary outcomes (admission to the intensive care unit—ICU, need for invasive mechanical ventilation—IMV, and in-hospital deaths) were analyzed. In addition to the patterns of occurrence of comorbidities, the adjusted relative risks (aRRs) for death were detailed in each region.

The data analysis showed that there were significant differences in the profile and outcomes of COVID-19 patients hospitalized in different regions. It was shown that the north region had the lowest vaccination rates for all age groups, and the north and northeast regions were the most affected by IMV requirements and in-hospital deaths in all age groups. Furthermore, the comorbidity analysis demonstrated that the pattern of comorbidities in the north and northeast regions in the younger age group was similar but different from the central–southern regions of Brazil.

## 2. Materials and Methods

### 2.1. Study Setting

Brazil is divided geopolitically into five major regions, named the north region (with seven federative units), the northeast region (with nine federative units), the midwest region (with four federative units), the southeast region (with four federative units), and the south region (with three federative units) (Figure S1). Since the pandemic’s beginning until December 2022, Brazil has accumulated 36,331,281 cases and more than 690,000 deaths, with significantly high rates of COVID-19 [17]. The country faced four successive waves due to the prevalence of the ancestral SARS-CoV-2, followed by the variants of concern Gamma/P.1, Omicron and subvariants Omicron BA.4/BA.5 [1,18]. 

Brazil created one of the largest public health systems in the world in 1990, named the Unified Health System (SUS), which offers universal, comprehensive, and no-charge health services to the Brazilian population [19]. In addition, SUS is also responsible for vaccination campaigns for vaccine-preventable diseases. However, in a country with continental dimensions and significant demographic, socioeconomic, and cultural differences, this system does not operate homogeneously in all regions, including having insufficient ICU bed units [20,21]. Social and ethnic inequalities and hospital infrastructure among Brazilian regions also contributed to the regional heterogeneity of in-hospital mortality of COVID-19 in Brazil [22,23]. A study carried out at the beginning of the COVID-19 pandemic in Brazil showed vast regional differences in hospital resources (ICU beds and equipment such as respirators/ventilators, ECG monitors, defibrillators, infusion pumps, and CT scanners), with low availability in the north and northeast regions and a greater concentration of resources in the south and southeast regions [24]. 

Variations in the percentage of the vaccinated population in the different Brazilian states are also observed regarding adherence to vaccination. For example, data obtained in 2023 showed that the booster dose (third dose) was applied to 27.26% and 28.4% of the Roraima (north region) and Pará (northeast region) population, respectively, while 86.42% and 70.33% of the Minas Gerais and Rio de Janeiro (southeast region) population, respectively, received a booster dose (Table S2).

### 2.2. Data Extraction

Data from COVID-19 patients hospitalized from 1 January 2022 to 31 December 2022 were extracted from the SIVEP-Gripe database (Information System for Epidemiological Surveillance of Influenza) available from the Ministry of Health (Brazil, MS, 2022). The SIVEP-Gripe database provides de-identified data on hospitalized patients and deaths due to severe acute respiratory syndromes in Brazil. Therefore, the data used in this study do not involve patients directly, and ethical committee approval is not required. The data refer to the database of 4 April 2023. Only patients confirmed by the quantitative reverse-transcriptase polymerase chain reaction technique (RT-PCR) molecular test or immunological diagnosis (antibodies or antigen test) for SARS-CoV-2, aged 18 years or older, were included in the analysis. The constructed dataset encompassed epidemiological information (age, gender, region of notification, and vaccine status) and comorbidities (asthma, cardiovascular disease, diabetes, kidney disease, hematological disease, immunosuppression, liver disease, neuropathy, pneumopathy, and obesity). In addition, it included ICU admission data, invasive and non-invasive ventilation needs, and evolution (cure or death).

In the data preparation process, new variables were created to represent the age group (18–39, 40–59, 60–79, and ≥80 years) based on age and number of vaccine doses (based on the dates of the first and second doses and booster dose). Patients were considered vaccinated 14 or more days after the last dose and symptom onset. Patients with inconsistent vaccination data were not considered. The outcome variables have missing data between [7–12%], except the evolution, which is complete. The comorbidities have between [14–29%] of missing data. Thus, the percentage analyses of comorbidities were performed considering a lower value of n, without the imputation of values. 

### 2.3. Statistical Analysis

Frequency analyses (%) were performed for categorical variables. Logistic regression models were used to calculate adjusted odds ratios (aORs) with 95% confidence intervals. Poisson regression models with robust variance estimator were used to calculate relative risks. Adjusted relative risks (aRR) and 95% confidence intervals were reported. Analysis of vaccine intake and unfavorable outcomes were performed to investigate the differences among the five regions. Odds ratios were reported to estimate the chance of having 0 doses, 1 dose, 2 doses, or 3 doses, comparing regions. The relative risk estimated the risk of adverse outcomes in the five regions. Additionally, relative risks for death were calculated stratified by regions. Adjusted models included the following variables: gender, age group, comorbidities, vaccination status, ICU admission, and IMV requirement. Missing information regarding comorbidities was assumed as absent in this analysis. All analyses were performed using Python (version 3.10.6) and the statsmodel module (version 0.14.0). A significance level of 0.05 was considered.

## 3. Results

### 3.1. Description of the Participants

Here, we analyzed clinical–epidemiological data from 115,638 Brazilian COVID-19 patients hospitalized during 2022 (prevalence of Omicron variant/subvariants), stratified by region and age group (Table 1). In the southeast, south, midwest, and northeast regions, approximately 70% of hospitalized patients were ≥60 years old, while in the north region, this percentage was lower, corresponding to 62%.

**Figure 1:**
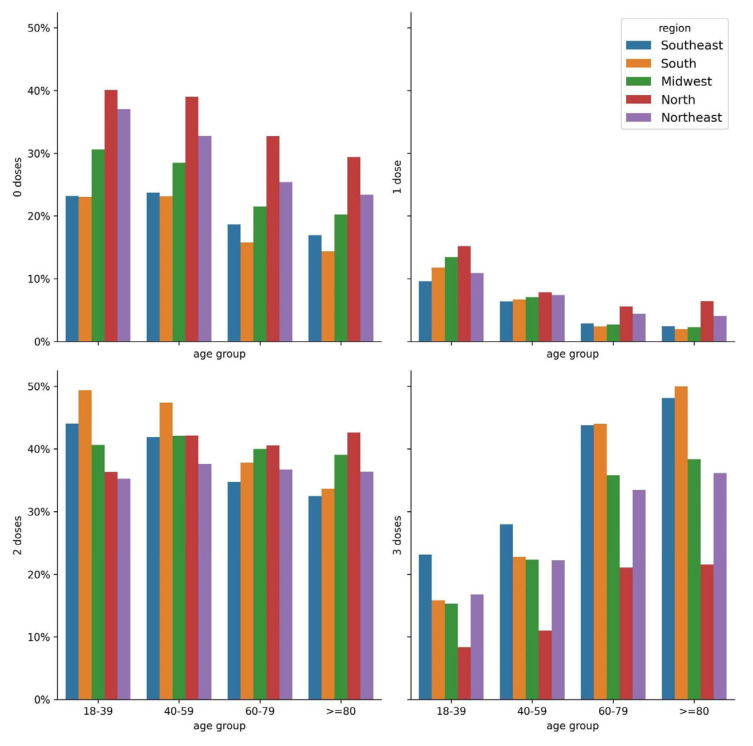
Percentage of patients hospitalized during the prevalence of the Omicron variant/subvariants (1 January 2022–31 December 2022) vaccinated with 1, 2, and 3 doses and unvaccinated stratified by region and age group.

Regarding vaccine status (Figure 1, Table S1), it was observed that the southeast and south regions had a higher percentage of hospitalized patients vaccinated with three doses in all age groups. It is important to highlight that these regions had greater vaccination coverage with three doses, with approximately 68% of the population receiving the third dose (Table S2), possibly numerically reflecting a greater probability of hospitalization according to the size of the vaccinated population. In contrast, the north region had higher rates of unvaccinated hospitalized patients in all age groups; showing increased odds for the 18–39 (aOR = 2.17, 95% CI = (1.80–2.61), *p* < 0.001), 40–59 (aOR = 2.07, 95% CI = (1.76–2.43), *p* < 0.001), 60–79 (aOR = 2.04, 95% CI = (1.80–2.31), *p* < 0.001), and ≥80 (aOR = 1.95, 95% CI = (1.66–2.28), *p* < 0.001) age groups compared to patients from the southeast region (Table S1). Accordingly, vaccination coverage in the north region was the lowest with the first dose (75.49%) (Table S2), consequently increasing the chances of hospitalization of unvaccinated individuals. The region with the lowest number of hospitalized patients vaccinated with three doses was also the north, with approximately half the percentage of the southeast region in all age groups, presenting reduced odds for the 18–39 (aOR = 0.31, 95% CI = (0.22–0.42), *p* < 0.001), 40–59 (aOR = 0.31, 95% CI = (0.25–0.40), *p* < 0.001), 60–79 (aOR = 0.35, 95% CI = (0.31–0.40), *p* < 0.001), and ≥80 (aOR = 0.30, 95% CI = (0.25–0.360) *p* < 0.001) age groups (Table S1). These data agree with the lower percentage of the population in the north region being vaccinated with the booster dose (41.02%) (Table S2). A similar profile to the north region was also observed in the northeast and midwest regions, with increased odds for unvaccinated patients and reduced odds for booster-vaccinated patients in all age groups compared to patients in the southeast region (Table S1). It is important to emphasize that patients in the older age groups tended to have more doses, possibly due to the National Vaccination Plan (PNI) scheme, which included the elderly as a priority group to receive vaccine doses (Figure 1, Table S1). Notably, the percentage of hospitalized patients vaccinated with only one dose was lower in all regions compared with the other three groups of 0, 2, and 3 doses (Figure 1), suggesting a smaller percentage of individuals in this condition in the general Brazilian population, and that most of the vaccination-adherent population completed the dosing schedule.

### 3.2. Analysis of Outcomes and Comorbidities

**Figure 2:**
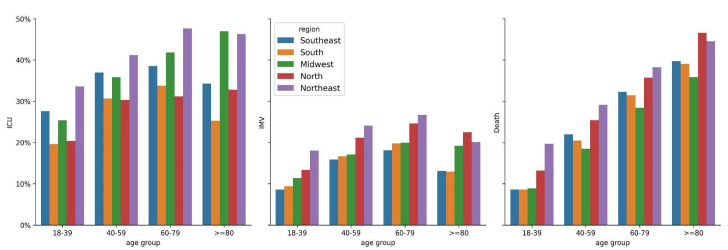
Outcomes of hospitalized patients during the prevalence of the Omicron variant/subvariants (1 January 2022–31 December 2022). Percentage values by region and age group.

The adverse outcomes among hospitalized COVID-19 patients are shown in Figure 2. ICU admission ranged from ~20% to ~48%, depending on region and age group. The region with the highest ICU admission rates was the northeast, with increased risks for the 18–39 (aRR = 1.13, 95% CI = (1.03–1.24), *p* = 0.011), 40–59 (aRR = 1.11, 95% CI = (1.05–1.18), *p* < 0.005), 60–79 (aRR = 1.23, 95% CI = (1.19–1.28), *p* < 0.001), and ≥80 (aRR = 1.36, 95% CI = (1.30–1.41), *p* < 0.001) age groups compared to the southeast region (Table S3). Notably, the northeast region had lower complete (79.28%) and third-dose (68.54%) vaccination coverage than the southeast region (84.55% and 68.54%, respectively) (Table S2), with similar age distribution (Table 1). In contrast, the north and south regions had the lowest occurrence of ICU admissions in all age groups. The north region had reduced risks for the 18–39 (aRR = 0.72, 95% CI = (0.60–0.86), *p* < 0.001), 40–59 (aRR = 0.83, 95% CI = (0.74–0.94), *p* < 0.005), and 60–79 (aRR = 0.82, 95% CI = (0.75–0.89), *p* < 0.001) age groups, and did not show a significant difference in the age group of ≥80 (aRR = 0.97, 95% CI = (0.87–1.07), *p* = 0.518) compared to the southeast region (Table S3). Patients from the south region had significantly low ICU admissions (Figure 2, Table S3), as well as a low percentage of unvaccinated patients and a higher percentage of patients receiving two or three vaccine doses.

Regarding the IMV requirement, the north and northeast regions had the highest occurrences in all age groups. Compared to the southeast region, the north regions showed increased risks for IMV requirement in the 40–59 (aRR = 1.33, 95% CI = (1.13–1.56), *p* < 0.005), 60–79 (aRR = 1.35, 95% CI = (1.21–1.51), *p* < 0.001), and >=80 (aRR = 1.66, 95% CI = (1.43–1.93), *p* < 0.001) age groups (Table S3). Similarly, the northeast region showed increased risks for IMV in all age groups, compared to the southeast region: 18–39 (aRR = 1.78, 95% CI = (1.51–2.10), *p* < 0.001), 40–59 (aRR = 1.49, 95% CI = (1.35–1.63), *p* < 0.001), 60–79 (aRR = 1.46, 95% CI = (1.37–1.54), *p* < 0.001), and ≥80 (aRR = 1.52, 95% CI = (1.41–1.63), *p* < 0.001) (Table S3). The south region was more similar to the southeast region, with no significant differences (Figure 2, Table S3).

The analysis of the worst outcome (in-hospital death) for patients with COVID-19 showed significant differences among regions. In particular, the north and northeast regions had the highest occurrences of in-hospital deaths in all age groups. Compared to the southeast region, significantly increased risks for death in the 18–39 (aRR = 1.81, 95% CI = (1.56–2.11), *p* < 0.001), 40–59 (aRR = 1.28, 95% CI = (1.18–1.38), *p* < 0.001), 60–79 (aRR = 1.16, 95% CI = (1.12–1.21), *p* < 0.001), and ≥80 (aRR = 1.10, 95% CI = (1.06–1.14), *p* < 0.001) age groups were shown by the northeast region (Table S3). The north region presented increased risks for death in the 60–79 (aRR = 1.08, 95% CI = (1.00–1.17), *p* = 0.041) and >=80 (aRR = 1.11, 95% CI = (1.03–1.20), *p* = 0.006) age groups (Table S3). A particularly notable difference occurred in the age group of 18–39 when comparing the northeast and southeast regions, where the percentage of deaths in the northeast exceeded 2× the value in the southeast (Figure 2, Table S3). Overall, the results showed that the IMV requirements and the in-hospital deaths were higher among patients from the north and northeast regions, which had lower full/third-dose vaccine coverage compared to those hospitalized in the southeast region, with high rates of full (84.55%) and third-dose (68.54%) vaccine coverage (Table S2).

**Figure 3:**
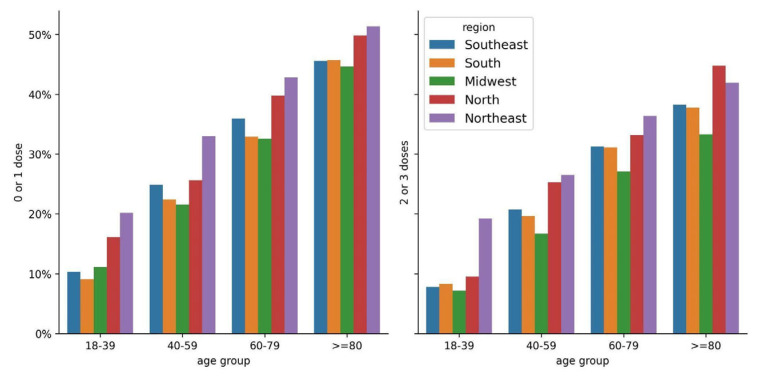
In-hospital deaths of patients hospitalized during the prevalence of Omicron variant/subvariants (1 January 2022–31 December 2022). Percentage values by region, age group, and number of doses of COVID-19 vaccine.

Considering the differences in the vaccination status and poor outcomes of patients in different regions, we evaluated the death rates in the subgroups of patients vaccinated with zero or one dose and two or three doses in different age groups for the five regions. Figure 3 shows a downward death trend for patients vaccinated with two or three doses. Overall, patients vaccinated with two or three doses showed a lower risk of death, with statistically significant results in the following cases: 18–39 (aRR = 0.78, 95% CI = (0.64–0.97), *p* = 0.022), 40–59 (aRR = 0.80, 95% CI = (0.73–0.88), *p* < 0.001), 60–79 (aRR = 0.85, 95% CI = (0.81–0.89), *p* < 0.001), and ≥80 (aRR = 0.83, 95% CI = (0.79–0.87), *p* < 0.001) age groups in the southeast region; 40–59 (aRR = 0.80, 95% CI = (0.68–0.93), *p* < 0.005) and ≥80 (aRR = 0.76, 95% CI = (0.70–0.82), *p* < 0.001) age groups in the south region; 60–79 (aRR = 0.81, 95% CI = (0.72–0.92), *p* < 0.005) and ≥80 (aRR = 0.80, 95% CI = (0.71–0.91), *p* < 0.001) age groups in the midwest region; 18–39 (aRR = 0.42, 95% CI = (0.22–0.78), *p* = 0.007) age group in the north region; 40–59 (aRR = 0.74, 95% CI = (0.63–0.88), *p* < 0.001), 60–79 (aRR = 0.87, 95% CI = (0.79–0.97), *p* < 0.010), and ≥80 (aRR = 0.79, 95% CI = (0.72–0.86), *p* < 0.001) age groups in the northeast region (Figure 3, Table S4).

**Figure 4:**
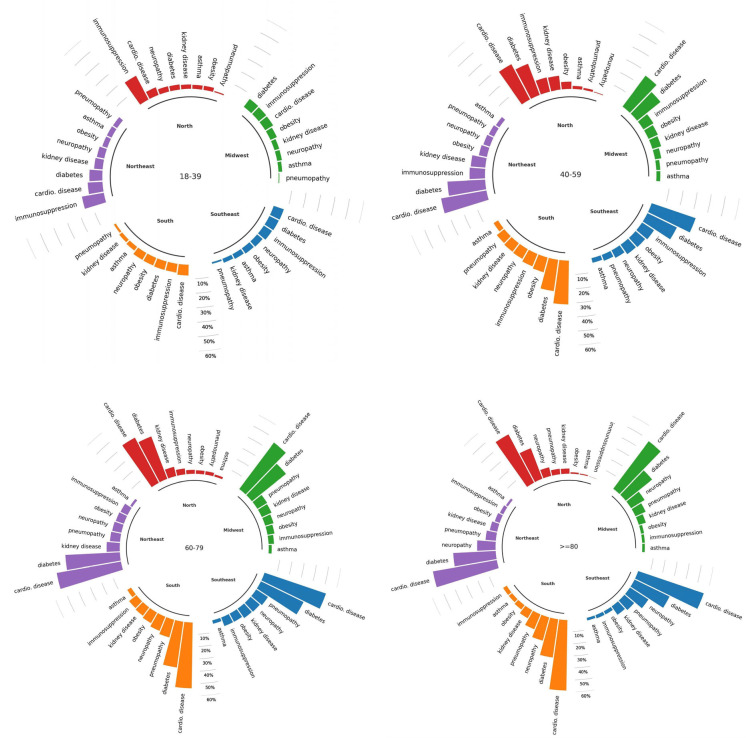
Comorbidities presented by patients hospitalized with COVID-19 during the prevalence of Omicron variant/subvariants (1 January 2022–31 December 2022) by age group and region.

We evaluated other variables that significantly impact outcomes because they are risk factors. Figure 4 shows the occurrences of the main comorbidities presented by hospitalized COVID-19 patients in terms of age group and region. There is an increasing trend in comorbidities as the age group increases. Cardiovascular disease, diabetes, and immunosuppression are the three most common comorbidities in patients aged 18–39 years in the south, southeast, and midwest regions, while pneumopathy is the least frequent. A different pattern is observed in the north and northeast regions, where immunosuppression appears more frequently, 19.21% and 16.04%, respectively, compared to patients from other regions (with approximately 7%). In summary, regardless of age or region, cardiovascular disease and diabetes are the most frequent comorbidities among hospitalized COVID-19 patients (Figure 4, Table S5).

### 3.3. Risk Factors for In-Hospital Death by Region

**Figure 5:**
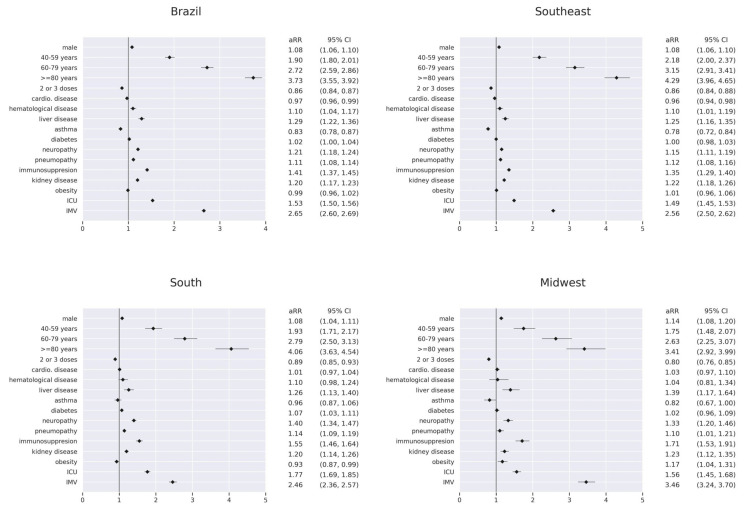

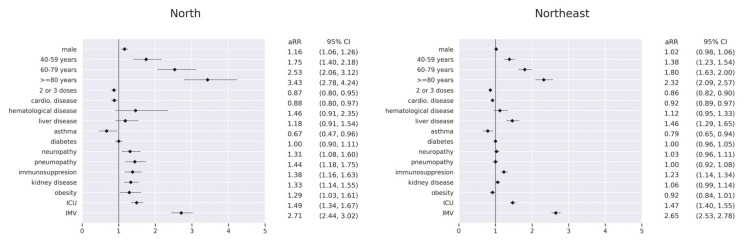
Adjusted relative risks of death presented by patients hospitalized with COVID-19 during the prevalence of Omicron variant/subvariants (1 January 2022–31 December 2022) stratified by region.

The analysis of aRRs showed that the risk of death increased significantly as the age group of COVID-19 patients increased in any of the five Brazilian regions compared to the 18–39 age group. Notably, the relative risks of death for COVID-19 patients in the 40–59 (aRR = 1.38, 95% CI = (1.23–1.54), *p* < 0.001), 60–79 (aRR = 1.80, 95% CI = (1.63–2.00), *p* < 0.001), and ≥80 (aRR = 2.32, 95% CI = [2.09–2.57], *p* < 0.001) age groups hospitalized in the northeast region were significantly lower compared with patients from other regions. In contrast, it is essential to highlight that patients aged 18–39 from the northeast showed a different pattern in lethality rates compared to other regions. This can explain the difference for the northeast region. For the gender variable, there was a slight tendency towards a higher risk of death among males in all regions. Full/booster (two or three doses) vaccination significantly reduced the risk of death for patients hospitalized with COVID-19 in Brazil (aRR = 0.86, 95% CI = (0.84–0.87), *p* < 0.001) in all regions: southeast (aRR = 0.86, 95% CI = (0.84–0.88), *p* < 0.001); south (aRR = 0.89, 95% CI = (0.85–0.93), *p* < 0.001); midwest (aRR = 0.80, 95% CI = (0.76–0.85), *p* < 0.001); north (aRR = 0.87, 95% CI = (0.80–0.95), *p* < 0.005); northeast (aRR = 0.86, 95% CI = (0.82–0.90), *p* < 0.001) (Figure 5).

Regarding comorbidities, region-disaggregated data showed that immunosuppressed COVID-19 patients from all regions were at increased risk of death, with a more substantial risk in the midwest (aRR = 1.71, 95% CI = (1.53–1.91), *p* < 0.001). In addition, hospitalized patients with liver disease, neuropathy, pneumopathy, and kidney disease in most regions were at significantly increased risk of death. In contrast, asthma patients from the southeast (aRR = 0.78, 95% CI = (0.72–0.84), *p* < 0.001), north (aRR = 0.67, 95% CI = (0.47–0.96), *p* < 0.05), northeast (aRR = 0.79, 95% CI = (0.65–0.95), *p* < 0.05), and midwest (aRR = 0.82, 95% CI = (0.67–1.00), *p* < 0.05) regions had a significantly lower risk of death (Figure 5).

Finally, the aRRs for in-hospital deaths of patients who required ICU and IMV were significantly high in all five Brazilian regions, consistent with the severity of COVID-19. The relative risk was higher for patients admitted to the ICU in the south region (aRR = 1.77, 95% CI = (1.69–1.85), *p* < 0.001) and for patients requiring IMV in the midwest region (aRR = 3.46, 95% CI = (3.24–3.70), *p* < 0.001) (Figure 5).

Collectively, the data analysis showed significant differences in vaccination status in the different Brazilian regions, especially for the north region, which had the lowest booster dose rates and the highest rates of unvaccinated patients for all age groups. Regarding unfavorable outcomes, the northeast region had the highest rates of patients admitted to the ICU for most age groups, while the north and northeast regions were the most affected by IMV requirements and in-hospital deaths in all age groups.

## 4. Discussion

The COVID-19 pandemic, which has affected countries on all continents since 2020, involved different sectors of society working toward its containment. Non-pharmaceutical interventions, such as social distancing, the use of masks, hand and surface hygiene, and even lockdown, were essential to reducing the transmission of the SARS-CoV-2 etiological agent [16]. However, vaccines produced in record time and approved for emergency use were the primary intervention to prevent severe symptomatic cases, hospitalization, and deaths. On the other hand, the high number of individuals with COVID-19 throughout the pandemic has driven the emergence of new coronavirus variants, capable of escaping the immunity induced by a previous infection or by vaccines manufactured against the ancestral virus reported in Wuhan (China) [25,26,27]. Furthermore, it was found that after 4–6 months of the complete vaccination schedule (two doses/single dose), there is a waning immunity conferred by the vaccines [28,29]. Such factors have contributed to the occurrence of reinfection and vaccine breakthrough infection (occurrence of SARS-CoV-2 infection 14 or more days after completing the primary vaccine schedule) [12]. In the absence of vaccines for variants, the administration of booster doses was the strategy used, which proved capable of restoring immunity and protecting against severe disease and death [30,31]. 

Vaccine coverage did not occur homogeneously in all Brazilian regions. Brazil initially made available the vaccines CoronaVac (Sinovac/Instituto Butantan), Vaxzevria (AstraZeneca/Oxford/Fiocruz), Comirnaty (Pfizer/Wyeth), and Janssen (Johnson&Johnson), that were administered in a homologous or heterologous regime. Priority was given to Comirnaty (Pfizer/Wyeth) for booster doses. The distribution of the vaccine types according to manufacturer depended on acquisition by the Brazilian government and availability for delivery to different regions [32]. Vaccination coverage was higher with the first dose and decreased with the second and third doses (booster) in all regions. The percentage of individuals vaccinated with the first dose was higher in the southeast and south regions, with approximately 87% of the vaccinated population, and lower in the north region (75.49%). Vaccination coverage with the second dose was also higher in the southeast and south regions, with around 85% of the population vaccinated, and lower in the north region (67.86%). Likewise, vaccination coverage with the third dose was also higher in the southeast (68.54%) and south (67.91%) and lower in the north (41.02%) (Table S2). 

The present study aimed to evaluate the epidemiological and clinical profile of hospitalized COVID-19 patients in the five macro-regions of Brazil in order to identify the most vulnerable subgroups during the predominance of the Omicron variant/subvariants hitting Brazil throughout 2022. In agreement with previous studies [10,21,22,33], elderly people aged 60 years and older with comorbidities (mainly cardiovascular disease and diabetes) are more likely to be hospitalized from COVID-19. Regarding vaccine status, it was possible to observe a significantly high rate of patients with zero to one dose who were hospitalized, as well as a significant rate of patients, mainly elderly, with complete/booster vaccination, suggesting a decline in vaccine-induced immunity over time and/or the escape of the Omicron variant/subvariants. Although with variations between regions, these data demonstrated the importance of the complete/booster vaccination schedule in reducing in-hospital deaths in all the age groups analyzed.

A study evaluated the clinical characteristics and outcomes of vaccinated and unvaccinated Brazilian patients with cardiovascular diseases hospitalized during the prevalence of the Omicron variant and found that the factors of youngest, male, diarrhea, ICU admission, invasive ventilation requirement, and death were associated with unvaccinated patients [34]. Although it did not prevent infection during the Omicron variant prevalence, another study highlighted the protective effect of complete and booster vaccination, even with inactivated vaccines, compared to those unvaccinated [35]. However, booster vaccine adherence, mainly the booster dose with the bivalent vaccine, has been low [36], even though bivalent COVID-19 booster doses have been shown to be effective in protecting against infection and death during BA.4/BA.5 circulation [37]. In line with these studies, we also demonstrated the importance of the full/booster vaccination schedule in protecting against severe COVID-19 outcomes.

In the north and northeast regions, with lower vaccination coverage, there was a higher rate of IMV requirements and deaths in all age groups. Importantly, in the north and northeast regions, where there was a higher death rate in the 18–39 age group, a higher percentage of immunosuppressed patients was also observed, exceeding those with cardiovascular disease and diabetes. These relevant data deserve further attention, as the humoral response among immunosuppressed individuals can decline significantly six months after the second dose [38].

The relative risk analyses showed that, in addition to advanced age, underlying medical conditions, especially immunosuppression, increased the risk of unfavorable outcomes for hospitalized COVID-19 patients. These results are consistent with a recent study performed with Brazilian hospitalized COVID-19 patients [39]. Relative risk analyses for death also showed relevant results according to the regions since Brazil has continental dimensions and significant socioeconomic, cultural, and public health infrastructure differences.

Regarding the ICU, the risk of patient admission was higher in the northeast region, possibly because they were patients of the subgroup with lower full/booster vaccination rates than patients in the other regions; therefore, they were more susceptible to severe disease outcomes. In contrast, patients from the north region had a lower risk of admission to the ICU, although the rates of patients with full/booster vaccination were low. A possible explanation is that except for immunosuppression, hospitalized patients of all age groups in this region had fewer comorbidities (Table S5) and, therefore, were less likely to require ICU admission. Regarding the south region, considering all age groups, approximately 75% of hospitalized patients were full/booster vaccinated (Table S5) and may have been protected by additional vaccine doses. We can infer, therefore, that the clinical–epidemiological profile and vaccine status of hospitalized COVID-19 patients, in addition to the waning immunity over time, may influence the disease outcome.

The present study demonstrated that complete/booster vaccination is essential in reducing severe COVID-19 cases and deaths. Furthermore, a recent study showed that recurrent infections, particularly after the emergence of the Omicron variant, can cause long-term COVID, and vaccination can protect against post-acute sequelae [40]. Therefore, campaigns encouraging the adherence of the population to vaccination in order to expand vaccination coverage in the country are critical to fight the disease, especially in the north and northeast regions. Due attention should also be given to mainly immunosuppressed individuals, in addition to the elderly, who have lower immunity and are more likely to develop severe disease.

The main strength of our study is its large sample size involving 115,638 hospitalized COVID-19 patients in 2022, during the prevalence of the Omicron variant/subvariants. Another important aspect is the stratification of Brazil’s macro-regions, highlighting the population’s asymmetric characteristics. The study’s main limitation is the lack of information in the database of non-hospitalized patients who represent less severe cases. Another limiting aspect is the use of registry data, which were not designed to answer a specific study question; therefore, variables and potential confounders could not have been collected.

## Figures and Tables

**Table 1:** Patients hospitalized during the prevalence of the Omicron variant/subvariants (1 January 2022–31 December 2022). Absolute values and percentages by region and age group.

Age Group	Southeast	South	Midwest	North	Northeast	All
18–39 years	5788 (9.11%)	2785 (10.54%)	1348 (13.02%)	539 (16.68%)	1220 (10.10%)	11,680 (10.10%)
40–59 years	10,166 (15.99%)	4405 (16.67%)	1997 (19.30%)	700 (21.66%)	2061 (17.06%)	19,329 (16.71%)
60–79 years	25,223 (39.69%)	10,967 (41.50%)	4046 (39.09%)	1213 (37.54%)	4641 (38.42%)	46,090 (39.86%)
≥80 years	22,374 (35.21%)	8271 (31.30%)	2958 (28.58%)	779 (24.11%)	4157 (34.41%)	38,539 (33.33%)
**All**	**63,551 (100%)**	**26,428 (100%)**	**10,349 (100%)**	**3231** **(100%)**	**12,079 (100%)**	**115,638 (100%)**

## Data Availability

The data used in this study are publicly available at: https://opendatasus.saude.gov.br/sq/dataset/srag-2021-a-2023 (accessed on 4 April 2023).

## Supplementary Materials

The following supporting information can be downloaded at: https://www.mdpi.com/article/10.3390/vaccines11101568/s1, Supplementary Figure S1. Brazilian Regions. Brazil is divided into five major regions by the Brazilian Institute of Geography and Statistics (IBGE). The federative units are grouped according to geographic, social and economic factors. Generated with Python (3.10.6), pandas (1.5.3), matplotlib (3.7.1), and geobr (0.2.0). Supplementary Table S1. Number of vaccine doses in hospitalized patients during the prevalence of the Omicron variant and its subvariants (1 January 2022–31 December 2022). Percentage values by region and age group. Adjusted odds ratio (aOR) estimates the chance of having 0 doses, 1 dose, 2 doses, or 3 doses comparing regions (reference = southeast). Models were adjusted for gender and comorbidities. Supplementary Table S2: Percentage of the population vaccinated with 1, 2 or 3 doses in the different Brazilian states and regions until the beginning of March/2023. Supplementary Table S3. Outcomes of hospitalized patients during the prevalence of the Omicron variant and its subvariants (1 January 2022–31 December 2022). Percentage values by region and age group. Adjusted relative risks (aRR) for unfavorable outcomes comparing regions (reference = southeast). Models were adjusted for gender, doses, and comorbidities. Supplementary Table S4. In-hospital death (%) in patients during the prevalence of the Omicron variant and its subvariants (1 January 2022–31 December 2022). Percentage values by region, age group and number of vaccine doses. Adjusted relative risks (aRR) for death between 2 or 3 doses and 0 or 1 dose (reference). Models were adjusted for gender and comorbidities. Supplementary Table S5. Comorbidities in hospitalized patients during the prevalence of the Omicron variant and its subvariants (1 January 2022–31 December 2022). Percentage values by region and age group.
