# Supplementary material for: Profile and Outcomes of Hospitalized COVID-19 Patients during the Prevalence of the Omicron Variant According to the Brazilian Regions: A Retrospective Cohort Study from 2022
Source: Vaccines (Basel). 2023 Oct 5;11(10):1568. doi: 10.3390/vaccines11101568 (PMC10610688; doi:10.3390/vaccines11101568)
Supplement: Supplementary file 1 [file vaccines-11-01568-s001.zip › vaccines-2564986-supplementary.pdf]

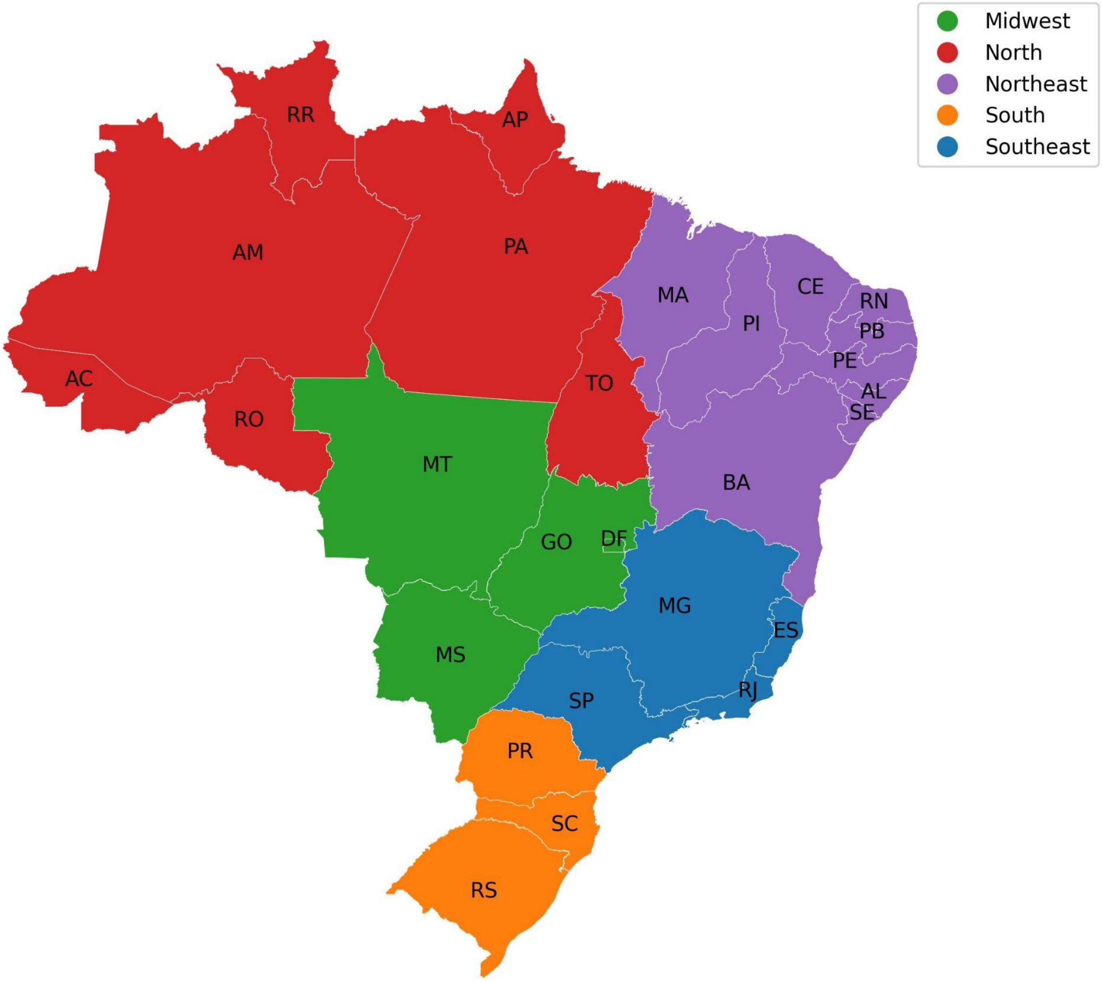

Supplementary Figure S1. Brazilian Regions. Brazil is divided into five major regions by the Brazilian Institute of Geography and Statistics (IBGE). The federative units are grouped according to geographic, social and economic factors. Generated with `Python` (3.10.6), `pandas` (1.5.3), `matplotlib` (3.7.1), and `geobr` (0.2.0).

Supplementary Table S1. Number of vaccine doses in hospitalized patients during the prevalence of the Omicron variant and its subvariants (01/01/2022 - 31/12/2022). Percentage values by region and age group. Adjusted odds ratio (aOR) estimates the chance of having 0 doses, 1 dose, 2 doses, or 3 doses comparing regions (reference=Southeast). Models were adjusted for gender and comorbidities.

age group	region	0 doses (%)	aOR (95% CI), p-value	1 dose (%)	aOR (95% CI), p-value	2 doses (%)	aOR (95% CI), p-value	3 doses (%)	aOR (95% CI), p-value
18-39	Midwest	30.64	1.46 (1.28-1.67), p<0.001	13.43	1.47 (1.23-1.76), p<0.001	40.65	0.86 (0.77-0.98), p=0.018	15.28	0.60 (0.51-0.71), p<0.001
	North	40.07	2.17 (1.80-2.61), p<0.001	15.21	1.62 (1.26-2.09), p<0.001	36.36	0.74 (0.62-0.89), p<0.005	8.35	0.31 (0.22-0.42), p<0.001
	Northeast	37.05	1.93 (1.69-2.20), p<0.001	10.90	1.13 (0.92-1.38), p=0.241	35.25	0.70 (0.62-0.80), p<0.001	16.80	0.67 (0.57-0.79), p<0.001
	South	23.05	1.00 (0.90-1.11), p=0.987	11.78	1.27 (1.10-1.47), p<0.005	49.34	1.23 (1.12-1.35), p<0.001	15.83	0.62 (0.55-0.70), p<0.001
	Southeast	23.22	1	9.61	1	44.04	1	23.13	1
40-59	Midwest	28.49	1.26 (1.14-1.41), p<0.001	7.06	1.11 (0.92-1.34), p=0.280	42.11	1.02 (0.92-1.12), p=0.710	22.33	0.74 (0.66-0.83), p<0.001
	North	39.00	2.07 (1.76-2.43), p<0.001	7.86	1.26 (0.94-1.67), p=0.119	42.14	1.01 (0.87-1.18), p=0.861	11.00	0.31 (0.25-0.40), p<0.001
	Northeast	32.75	1.57 (1.42-1.75), p<0.001	7.42	1.18 (0.98-1.42), p=0.078	37.60	0.83 (0.76-0.92), p<0.001	22.22	0.73 (0.66-0.82), p<0.001
	South	23.16	0.97 (0.89-1.05), p=0.417	6.70	1.05 (0.91-1.21), p=0.495	47.38	1.25 (1.16-1.34), p<0.001	22.77	0.76 (0.70-0.82), p<0.001
	Southeast	23.71	1	6.39	1	41.89	1	28.01	1

60-79	Midwest	21.50	1.16 (1.07-1.26), p<0.001	2.72	0.94 (0.77-1.16), p=0.573	39.99	1.26 (1.17-1.35), p<0.001	35.79	0.73 (0.68-0.78), p<0.001
	North	32.73	2.04 (1.80-2.31), p<0.001	5.61	2.01 (1.55-2.59), p<0.001	40.56	1.29 (1.15-1.45), p<0.001	21.10	0.35 (0.31-0.40), p<0.001
	Northeast	25.43	1.48 (1.38-1.60), p<0.001	4.42	1.58 (1.35-1.86), p<0.001	36.69	1.09 (1.02-1.16), p=0.012	33.46	0.65 (0.61-0.69), p<0.001
	South	15.82	0.83 (0.78-0.88), p<0.001	2.42	0.84 (0.73-0.97), p=0.017	37.79	1.14 (1.09-1.19), p<0.001	43.98	1.00 (0.96-1.05), p=0.923
	Southeast	18.67	1	2.86	1	34.71	1	43.76	1
>=80	Midwest	20.25	1.20 (1.09-1.33), p<0.001	2.30	0.94 (0.72-1.21), p=0.610	39.08	1.34 (1.24-1.45), p<0.001	38.37	0.68 (0.63-0.74), p<0.001
	North	29.40	1.95 (1.66-2.28), p<0.001	6.42	2.76 (2.05-3.73), p<0.001	42.62	1.56 (1.35-1.80), p<0.001	21.57	0.30 (0.25-0.36), p<0.001
	Northeast	23.41	1.49 (1.37-1.61), p<0.001	4.09	1.73 (1.45-2.06), p<0.001	36.37	1.19 (1.11-1.28), p<0.001	36.13	0.61 (0.57-0.65), p<0.001
	South	14.40	0.84 (0.78-0.90), p<0.001	1.97	0.80 (0.67-0.96), p=0.016	33.67	1.05 (1.00-1.11), p=0.056	49.96	1.07 (1.02-1.12), p=0.011
	Southeast	16.95	1	2.43	1	32.49	1	48.13	1

Supplementary Table S2. Percentage of the population vaccinated with 1, 2 or 3 doses in the different Brazilian states and regions until the beginning of March/2023.

	**1 dose (%)**	**2 doses (%)**	**3 doses (booster) (%)**
**Brazil**	**83.15±6.55**	**78.00±8.73**	**54.62±14.72**
**North**			
Acre	77.36	69.54	49.48
Amapá	72.91	64.51	48.32
Amazonas	80.03	71.10	57.89
Pará	78.27	75.65	28.40
Rondônia	73.25	67.54	38.57
Roraima	72.85	59.01	27.26
Tocantins	73.74	67.67	37.24
**Mean±SD**	**75.49±2.99**	**67.86±5.22**	**41.02±11.39**
**Northeast**			
Alagoas	77.78	69.21	43.20
Bahia	77.95	72.42	53.64
Ceará	93.85	87.94	60.82
Maranhão	80.33	67.19	33.08
Paraíba	87.51	83. 75	57.09
Pernambuco	88.96	83.84	57.16
Piauí	96.17	88.00	75.34
Rio Grande do Norte	83.72	79.79	57.01
Sergipe	86.51	81.42	58.84
**Mean±SD**	**85.86±6.57**	**79.28±7.84**	**55.13±11.71**
**Southeast**			
Espírito Santo	85.29	79.94	54.96
Minas Gerais	86.56	86.40	86.42
Rio de Janeiro	84.35	81.48	70.33

São Paulo	93.04	90.39	62.45
**Mean±SD**	**87.31±3.93**	**84.55±4.77**	**68.54±13.47**
**South**			
Paraná	88.9	89.10	73.03
Rio Grande do Sul	86.19	85.21	73.76
Santa Catarina	86.0	83.72	56.94
**Mean±SD**	**87.03±1.62**	**86.01±2.78**	**67.91±9.51**
**Midwest**			
Distrito Federal	90.55	89.72	60.10
Goiás	82.98	78.02	65.20
Mato Grosso	80.42	75.10	43.12
Mato Grosso do Sul	79.68	78.29	48.04
**Mean±SD**	**83.41±4.98**	**80.28±6.46**	**54.11±10.27**

Data source: Cota, W. Monitoring the number of COVID-19 cases and deaths in Brazil at municipal and federative units level. Available from: https://coronavirusbra1.github.io/ (accessed on September 5, 2023).

Supplementary Table S3. Outcomes of hospitalized patients during the prevalence of the Omicron variant and its subvariants (01/01/2022 -12/31/2022). Percentage values by region and age group. Adjusted relative risks (aRR) for unfavorable outcomes comparing regions (reference=Southeast). Models were adjusted for gender, doses, and comorbidities.

age group	region	ICU (%)	aRR (95% CI), p-value	IMV (%)	aRR (95% CI), p-value	Death (%)	aRR (95% CI), p-value
18-39	Midwest	25.36	0.95 (0.86-1.05), p=0.312	11.38	1.32 (1.10-1.59),p<0.005	8.90	1.02 (0.85-1.22), p=0.856
	North	20.33	0.72 (0.60-0.86), p<0.001	13.33	1.29 (1.00-1.67), p=0.055	13.17	1.17 (0.92-1.49), p=0.188
	Northeast	33.57	1.13 (1.03-1.24), p=0.011	18.04	1.78 (1.51-2.10), p<0.001	19.67	1.81 (1.56-2.11), p<0.001
	South	19.64	0.73 (0.67-0.80), p<0.001	9.42	1.11 (0.96-1.29), p=0.152	8.58	1.01 (0.88-1.17), p=0.880
	Southeast	27.58	1	8.61	1	8.64	1
40-59	Midwest	35.82	0.99 (0.93-1.06), p=0.785	17.07	1.09 (0.97-1.22), p=0.135	18.43	0.85 (0.77-0.93), p<0.005
	North	30.30	0.83 (0.74-0.94), p<0.005	21.15	1.33 (1.13-1.56), p<0.005	25.43	1.12 (0.98-1.27), p=0.102
	Northeast	41.20	1.11 (1.05-1.18), p<0.005	24.05	1.49 (1.35-1.63), p<0.001	29.11	1.28 (1.18-1.38), p<0.001
	South	30.64	0.82 (0.78-0.86), p<0.001	16.65	1.02 (0.94-1.11), p=0.592	20.48	0.91 (0.85-0.97), p=0.005
	Southeast	36.95	1	15.88	1	21.97	1
60-79	Midwest	41.82	1.10 (1.05-1.14), p<0.001	19.97	1.10 (1.03-1.19), p=0.006	28.42	0.88 (0.84-0.93), p<0.001
	North	31.13	0.82 (0.75-0.89), p<0.001	24.60	1.35 (1.21-1.51), p<0.001	35.70	1.08 (1.00-1.17), p=0.041
	Northeast	47.64	1.23 (1.19-1.28), p<0.001	26.68	1.46 (1.37-1.54), p<0.001	38.29	1.16 (1.12-1.21), p<0.001
	South	33.77	0.87 (0.84-0.90), p<0.001	19.74	1.08 (1.03-1.13), p<0.005	31.44	0.96 (0.93-0.99), p=0.018
	Southeast	38.55	1	18.09	1	32.26	1

>=80	Midwest	46.98	1.39 (1.33-1.45), p<0.001	19.20	1.44 (1.32-1.57), p<0.001	35.87	0.89 (0.84-0.93), p<0.001
	North	32.76	0.97 (0.87-1.07), p=0.518	22.48	1.66 (1.43-1.93), p<0.001	46.60	1.11 (1.03-1.20), p=0.006
	Northeast	46.29	1.36 (1.30-1.41), p<0.001	20.11	1.52 (1.41-1.63), p<0.001	44.53	1.10 (1.06-1.14), p<0.001
	South	25.24	0.73 (0.70-0.76), p<0.001	12.94	0.98 (0.92-1.05), p=0.579	39.06	0.98 (0.95-1.01), p=0.251
	Southeast	34.28	1	13.10	1	39.68	1

Supplementary Table S4. In-hospital death (%) in patients during the prevalence of the Omicron variant and its subvariants (01/01/2022 - 12/31/2022). Percentage values by region, age group and number of vaccine doses. Adjusted relative risks (aRR) for death between 2 or 3 doses and 0 or 1 dose (reference). Models were adjusted for gender and comorbidities.

region	age group	0 or 1 dose (%)	2 or 3 doses (%)	aRR [95% CI]	p-value
**Southeast**	18-39	10.32	7.82	0.78 [0.64-0.97]	0.022
	40-59	24.87	20.71	0.80 [0.73-0.88]	<0.001
	60-79	35.95	31.25	0.85 [0.81-0.89]	<0.001
	>=80	45.60	38.25	0.83 [0.79-0.87]	<0.001
**South**	18-39	9.07	8.32	0.92 [0.68-1.24]	0.581
	40-59	22.43	19.64	0.80 [0.68-0.93]	<0.005
	60-79	32.90	31.11	0.92 [0.84-1.01]	0.084
	>=80	45.72	37.76	0.76 [0.70-0.82]	<0.001
**Midwest**	18-39	11.11	7.16	0.76 [0.51-1.14]	0.189
	40-59	21.55	16.71	0.82 [0.65-1.03]	0.084
	60-79	32.55	27.10	0.81 [0.72-0.92]	<0.005
	>=80	44.68	33.30	0.80 [0.71-0.91]	<0.001
**North**	18-39	16.11	9.54	0.42 [0.22-0.78]	0.007
	40-59	25.61	25.27	0.85 [0.62-1.17]	0.329
	60-79	39.78	33.16	0.86 [0.71-1.03]	0.107
	>=80	49.82	44.80	0.92 [0.76-1.11]	0.375
**Northeast**	18-39	20.17	19.21	0.81 [0.61-1.08]	0.155
	40-59	32.97	26.52	0.74 [0.63-0.88]	<0.001
	60-79	42.82	36.36	0.87 [0.79-0.97]	0.010
	>=80	51.36	41.94	0.79 [0.72-0.86]	<0.001

Supplementary Table S5. Comorbidities in hospitalized patients during the prevalence of the Omicron variant and its subvariants (01/01/2022 -12/31/2022). Percentage values by region and age group.

region	age group	asthma	cardio. disease	diabetes	immunosuppression	kidney disease	neuropathy	obesity	pneumopathy
Southeast	18-39	4.52	8.19	7.97	7.51	3.73	5.75	5.27	2.01
	40-59	4.19	33.57	23.88	10.85	9.02	6.82	9.62	5.29
	60-79	3.68	53.43	39.28	8.30	10.18	10.65	8.40	12.17
	>=80	3.04	57.92	32.88	3.74	9.32	18.97	3.77	11.37
South	18-39	4.05	8.15	6.85	6.85	3.37	6.29	6.48	2.33
	40-59	4.51	31.59	23.05	10.28	8.29	8.66	11.52	7.55
	60-79	4.41	54.28	38.33	8.52	9.29	13.28	10.68	15.71
	>=80	4.34	59.00	32.69	4.29	8.43	20.82	5.27	14.21
Midwest	18-39	3.40	7.20	7.64	7.36	4.21	4.14	5.62	1.35
	40-59	3.57	27.90	21.93	9.13	7.26	5.30	8.03	3.63
	60-79	3.28	47.76	36.23	5.26	9.45	7.91	6.58	10.08
	>=80	2.96	49.60	28.67	3.06	6.88	13.86	3.31	10.02
North	18-39	3.57	6.29	4.26	19.21	3.80	4.47	3.56	1.66
	40-59	3.02	28.81	24.18	11.05	10.64	1.30	5.98	2.22
	60-79	2.57	42.18	37.54	5.65	8.51	4.07	3.73	3.63
	>=80	1.66	45.75	27.79	1.00	5.12	8.14	2.10	5.38
Northeast	18-39	4.40	10.99	9.73	16.04	6.74	6.03	4.44	4.20
	40-59	3.76	33.28	27.46	11.76	11.11	5.62	6.64	4.87
	60-79	2.77	53.77	45.10	7.13	11.51	8.45	7.78	9.02
	>=80	2.67	54.25	35.88	3.88	7.03	16.07	5.08	9.36
